# Molecular signatures of sanguinarine in human pancreatic cancer cells: *A large scale label-free comparative proteomics approach*

**DOI:** 10.18632/oncotarget.3231

**Published:** 2015-04-13

**Authors:** Chandra K. Singh, Satwinderjeet Kaur, Jasmine George, Minakshi Nihal, Molly C. Pellitteri Hahn, Cameron O. Scarlett, Nihal Ahmad

**Affiliations:** ^1^ Department of Dermatology, University of Wisconsin, Madison, WI, USA; ^2^ School of Pharmacy, University of Wisconsin, Madison, WI, USA; ^3^ William S. Middleton VA Medical Center, Madison, WI, USA

**Keywords:** pancreatic cancer, sanguinarine, quantitative proteomics, DUSP4, chemoprevention

## Abstract

Pancreatic cancer remains one of the most lethal of all human malignancies with its incidence nearly equaling its mortality rate. Therefore, it's crucial to identify newer mechanism-based agents and targets to effectively manage pancreatic cancer. Plant-derived agents/drugs have historically been useful in cancer therapeutics. Sanguinarine is a plant alkaloid with anti-proliferative effects against cancers, including pancreatic cancer. This study was designed to determine the mechanism of sanguinarine's effects in pancreatic cancer with a hope to obtain useful information to improve the therapeutic options for the management of this neoplasm. We employed a quantitative proteomics approach to define the mechanism of sanguinarine's effects in human pancreatic cancer cells. Proteins from control and sanguinarine-treated pancreatic cancer cells were digested with trypsin, run by nano-LC/MS/MS, and identified with the help of Swiss-Prot database. Results from replicate injections were processed with the SIEVE software to identify proteins with differential expression. We identified 37 differentially expressed proteins (from a total of 3107), which are known to be involved in variety of cellular processes. Four of these proteins (IL33, CUL5, GPS1 and DUSP4) appear to occupy regulatory nodes in key pathways. Further validation by qRT-PCR and immunoblot analyses demonstrated that the dual specificity phosphatase-4 (DUSP4) was significantly upregulated by sanguinarine in BxPC-3 and MIA PaCa-2 cells. Sanguinarine treatment also caused down-regulation of HIF1α and PCNA, and increased cleavage of PARP and Caspase-7. Taken together, sanguinarine appears to have pleotropic effects, as it modulates multiple key signaling pathways, supporting the potential usefulness of sanguinarine against pancreatic cancer.

## INTRODUCTION

Pancreatic cancer is one of the deadliest cancers because it is difficult to diagnose early, and is relatively resistant to treatment. The overall incidence of pancreatic cancer as well as mortality associated with it has not improved over the past several decades. It has an extremely poor prognosis, with ~20% one-year survival and a bleak ~3% five-year survival. In the United States alone, 46,420 new cases and 39,590 pancreatic cancer related deaths are predicted for year 2014 [1]. Since the conventional chemotherapy has not shown desired therapeutic outcome, it is crucial to identify novel mechanism-based modalities that can be effectively used alone or in conjunction with existing therapeutics for the management of this dreaded cancer.

From ancient time, natural products, including plant-alkaloids, have been a fertile source for anti-cancer drugs. Sanguinarine (13-methyl-[1,3]benzodioxolo[5,6-c]- 1,3-dioxolo[4,5-i]phenanthridinium) is a bioactive benzophenanthridine alkaloid found in plants of the Papaveraceae family, blood root plant *Sanguinaria canadensis*, and *Chelidonium majus*, and *Argemone Mexicana* [2]. Plants synthesize sanguinarine from dihydrosanguinarine through the action of dihydrobenzophenanthridine oxidase. Sanguinarine has been shown to possess broad spectrum pharmacological properties including anti-microbial, anti-oxidative and anti-inflammatory activities [2]. Several *in vitro* and *in vivo* studies have demonstrated sanguinarine's anti-cancer properties in variety of cancers [3–15]. We have previously demonstrated that sanguinarine imparts anti-proliferative effects in human epidermoid carcinoma (A431) cells without affecting normal cells (human epidermal keratinocytes) [10]. We have also demonstrated that sanguinarine imparts anti-proliferative effects against pancreatic cancer cells, AsPC-1 and BxPC-3, via modulation in Bcl-2 family proteins [9].

Therefore, sanguinarine has shown excellent developmental promise for treatment of cancer, including pancreatic cancer. This necessitates a need for a more in depth understanding of mechanism(s) of sanguinarine's action, which may be useful in multiple ways. First, identification of mechanistic signature of sanguinarine in pancreatic cancer cells may further validate if this alkaloid is a suitable candidate for anti-cancer drug development. Second, this may also help in identifying genes and/or protein targets modulated by sanguinarine that could be developed as surrogate biomarkers in preclinical studies and future clinical trials. Finally, this may also lead to discovery of novel targets for the management of pancreatic cancer.

Thus, the objective of this study was to decipher the molecular mechanism of the anti-proliferative effects of sanguinarine by interrogating the proteomics changes incurred by sanguinarine treatment in pancreatic cancer cells. For this purpose, we chose a label-free nano-ESI ultra high resolution mass spectrometry approach employing Q-Exactive hybrid quadrupole-Orbitrap mass spectrometer. Indeed, quantitative proteomics combined with bioinformatics is a powerful tool that can be used to reveal the complex molecular events in biological systems. The speed and efficiency of modern mass spectrometers allow data from thousands of peptides to be collected in a few hours. Database searching and post-processing can then be used to reveal quantitative changes in proteins from a broad array of biochemical and signaling pathways. In order to determine the molecular signatures associated with sanguinarine's anti-proliferative response, we subjected sanguinarine treated BxPC-3 pancreatic cancer cells to quantitative proteomics using SIEVE, a label-free relative quantitation strategy that uses rigorous statistics to quantitate LC-MS/MS peptide peaks. Label-free approaches are becoming more popular due to the vast improvements in instrumentation capabilities, as well as the relative lower cost of label-free experiments in comparison to stable isotope labelling such as Stable Isotope Labeling by Amino Acids in Cell Culture (SILAC) and Isobaric Tags for Relative and Absolute Quantification (iTRAQ) [16]. The Q-Exactive mass spectrometer is particularly well suited for label free quantitation due to its fast scanning speed and high resolving power. The SIEVE software tool calculates peptide ratios based on variation in the MS peak intensities between sample populations [16]. Peptide ratio data in SIEVE can be stringently filtered using multiple statistical models, including ratio, *p*-values (Fishers Combined Probability), and coefficient of variance scores between biological replicates which can be used to assess sample variability. SIEVE has an advantage over label-free spectral counting methods as it has the capability of quantitating lower abundant proteins/peptides which may show up in LC-MS/MS with relatively little MS/MS spectral data. In this study, we identified 37 differentially expressed proteins (from a total of 3107), which may be useful in creating a molecular signature of sanguinarine that may be significantly relevant to pancreatic cancer.

## RESULTS

### Effect of sanguinarine on proteome profile of human pancreatic cancer cells

As a first step to identify the effect of sanguinarine on global proteome changes, we determined the best effective dose regimen for sanguinarine treatment using two human pancreatic cancer cell lines BxPC-3 and MIA PaCa-2. BxPC-3 cells represent adenocarcinoma of moderate to poor differentiation with high angiogenic potential while MIA PaCa-2 are poorly differentiated carcinoma with low levels of pro-angiogenic factors [17]. Anti-proliferative effect of sanguinarine (0.1, 0.2, 0.5, 1.0, 2.0, 4.0 and 8.0 μM; for 24 and 48 h) in BxPC-3 and MIA PaCa-2 cells were analyzed using an MTT assay. Both cell lines showed a dose and time dependent decrease in OD570 following treatment with sanguinarine, indicating a decrease in cellular proliferation/metabolism (Supplementary Figure 1A and 1B). We further assessed the effect of sanguinarine (24 h treatment) against BxPC-3 and MIA PaCa-2 cells on the cell growth and viability using trypan blue exclusion assay, and clonogenic survival, where equal number of sanguinarine treated cells were allowed to grow for 14 days followed by staining with crystal violet, and noticed significant inhibition in all three parameters. The cell morphology, growth, viability and clonogenic survival data are shown in Supplementary Figure 2A, 2B and 2C. We observed that sanguinarine resulted in a significant reduction in cell growth at 1 and 2 μM concentrations. However, a significant decrease in viability was observed only at 2 μM concentration of sanguinarine (Supplementary Figure 2B). Collectively, our data show that sanguinarine at lower concentration (< 1 μM) did not have much effect whereas 2 μM seems to produce excessive cell killing in both BxPC-3 and MIA PaCa-2 cells. Thus, we selected 1 μM concentration of sanguinarine (for 24 h) in BxPC-3 cells for proteomics analysis.

**Figure 1:**
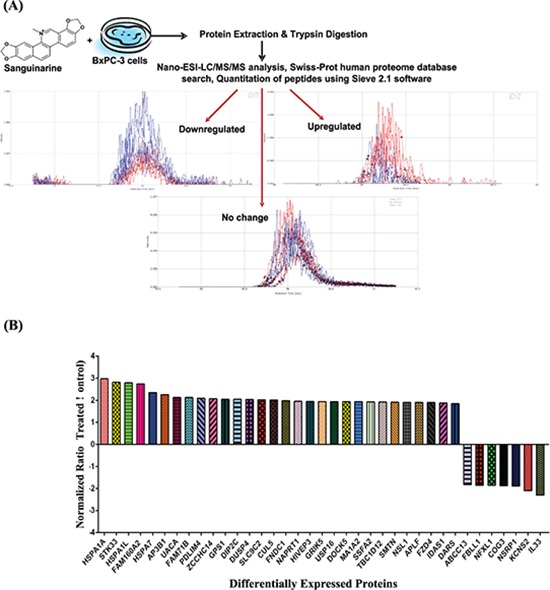
Effect of sanguinarine on proteome profile of BxPC-3 human pancreatic cancer cells **(A)** An illustration of experimental strategy for Nano-ESI-LC/MS/MS analysis and protein identification in sanguinarine treated BxPC-3 cells is shown. A typical results from analysis are presented showing an extracted ion chromatogram of control (blue) and sanguinarine-treated (red) peptides with examples of peptides showing down-regulation, up-regulation and no change. **(B)** Proteins showing > 1.8-fold change in abundance with sanguinarine treatment (95% confidence interval and *p*-value < 0.05) are shown. The data are representative of 3 biological and 2 technical replicates representing six sample for each group.

We performed a pre-validation experiment running duplicate injections of two biological replicate samples prepared identically. The pre-validation is important because despite advances in instrument technology and performance, data-dependent LC/MS/MS analysis of complex proteomics samples is still affected by under-sampling. This results run-to-run and sample-to-sample variability in the peptides selected for MS/MS analysis and which can be identified [18]. It is critical to have an understanding of the magnitude of this under-sampling so that sufficient replicate runs are done to have adequate statistical significance. When we compared the variability between duplicate injections of the same sample using a 2% false discovery rate (FDR) in our database search (lower than used in our actual analyses) we found 68% overlap. Comparing the overlap between biological replicates using 2% FDR we found a 66% overlap at the protein identification level (data not shown). Based on these, we elected to perform 6 replicate injections of each sample in our experimental protocol as shown in Figure 1A.

A crucial step in post-acquisition data processing is alignment of the base peak chromatograms. The SIEVE 2.1 uses a proprietary algorithm, Chromalign which in addition to aligning chromatogram regions of interest (in our case from retention time 30 min −122 min) also provides a measure of the quality of the alignment. Our data show excellent alignment as the scores of all the control and sanguinarine treated samples were ≥ 0.849, (Supplementary Figure 3A and B). SIEVE requires a score of 0.75 or above for valid quantitative analysis, scores above 0.85 are considered an excellent alignment. The output data were searched against the Swiss-Prot human proteome database with decoy using the Sequest HT search engine and further analyzed with the SIEVE 2.1 software to reveal the proteins with altered expression. This analysis resulted in a total of 3107 proteins identified at 0.05 Confidence Interval (CI) (Supplementary Table 1). The majority of these identified proteins showed no significant difference between treated and untreated samples. Less than 5% of the proteins (total 148 proteins) identified differed by > 1.5-fold (61 upregulated and 87 downregulated) (Supplementary Figure 4A). Further, the data of combined replicates compared at peptide and protein level show an overlap of 65% and 74%, respectively. The Venn diagram represented in Supplementary Figure 4B and 4C are generated using Proteome Discoverer (Thermo Fisher Scientific Inc.) identification software which re-groups the proteins if a peptide identified is 100% homologous in two or more different proteins. That's why the number of proteins identified by SIEVE 2.1 do not match with the protein numbers shown in Venn diagram. SIEVE is a quantification software and does not group proteins during the quantification because it has no way of knowing which protein the peptide belongs too. It gives the ratio data for all of the homologous proteins, thus inflate the protein numbers.

Out of 3107 identified proteins, we selected 37 proteins showing > 1.8 fold change with statistical significance (* p*-value < 0.05) (Table 1), for further analysis. The criteria for this selection (> 1.8 fold change) as a threshold was based on the fact that these proteins tend to numerically meet the principles of truly differential expressed proteins. Although some researchers have considered fold changes even less than 1.8-fold assuming adequate absolute expression levels, during validation it generally fail to reach statistical significance. Details about these 37 proteins which include protein ID, protein name, molecular weight, number of unique peptides, frames (defined rectangular region in the m/z vs. retention time plane), hits (ms/ms identification scans), % coverage of protein, average *p*-value and fold change upon sanguinarine treatment, are presented in Table 1. The fold change and direction of sanguinarine-modulated proteins are represented in Figure 1B.

**Table 1:** Details about modulated proteins upon sanguinarine treatment, showing >1.8 fold up- or down-regulation with statistical significance

S. No.	Protein ID	Gene Name	Protein Description	Molecular Weight	Unique Peptides	Frame Quantitated	His MS/MS	Sum Unique Peptides	Sum His MS/MS	% Coverage Control	% Coverage Control	Average *P*-value	Fold Change
1	P08107	HSPA1A	Heat shock 70 kDa protein 1A/1B	70,052	4	4	56	8	95	12.17%	20.28%	1.67E-03	2.974
2	Q9BYT3	STK33	Serine/threonine_protein kinase 33	57,831	2	1	5	3	8	7.73%	8.00%	5.80E-03	2.816
3	P34931	HSPA1L	Heat shock 70 kDa protein 1_like	70,375	2	2	28	6	80	9.86%	12.32%	3.91E-02	2.792
4	Q8N612	FAM160A2	FTS and Hook_interacting protein	105,568	2	2	2	2	5	1.09%	1.48%	1.82E-05	2.745
5	P48741	HSPA7	Putative heat shock 70 kDa protein 7	40,244	2	3	5	3	6	10.90%	6.54%	1.67E-03	2.352
6	O00203	AP3B1	AP_3 complex subunit beta_1	121,320	2	2	3	3	11	5.82%	8.55%	1.10E-03	2.259
7	Q9BZF9	UACA	Uveal autoantigen with coiled domains & ankyrin repeats	162,505	3	2	3	3	9	1.26%	2.56%	3.55E-03	2.131
8	Q8TC56	FAM71B	Protein FAM71B	64,756	2	2	5	4	7	6.61%	5.87%	1.20E-02	2.129
9	P50479	PDLIM4	PDZ and LIM domain protein 4	35,398	2	4	7	3	9	10.54%	5.98%	3.98E-02	2.093
10	Q8WYQ9	ZCCHC14	Zinc finger CCHC domain_containing protein 14	100,042	2	2	2	2	4	1.88%	1.21%	3.98E-02	2.07
11	Q13098	GPS1	CSN1_COP9 signalosome complex subunit 1	55,537	2	2	4	3	7	3.85%	4.23%	1.66E-02	2.051
12	Q9Y2E4	DIP2C	Disco_interacting protein 2 homolog C	170,767	2	2	5	3	8	3.17%	2.79%	4.88E-03	2.05
13	Q13115	DUSP4	Dual specificity protein phosphatase 4	42,953	2	2	4	3	12	5.47%	5.41%	4.88E-02	2.04
14	Q5TAH2	SLC9C2	Sodium/hydrogen exchanger 11	129,053	2	2	3	2	7	1.26%	1.26%	4.18E-02	2.019
15	Q93034	CUL5	Cullin_5	90,955	2	2	2	2	4	2.51%	2.12%	7.77E-03	2.012
16	Q4ZHG4	FNDC1	Fibronectin type III domain_containing protein 1	205,558	2	2	2	3	5	0.87%	0.95%	3.42E-03	1.985
17	Q6XQN6	NAPRT1	Nicotinate phosphoribosyltransferase	57,578	2	2	4	3	11	2.60%	3.98%	4.88E-02	1.952
18	Q5T1R4	HIVEP3	Transcription factor HIVEP3	259,465	2	2	3	3	8	0.97%	0.89%	3.42E-02	1.949
19	Q16478	GRIK5	Glutamate receptor_ ionotropic kainate 5	109,265	3	3	3	4	9	1.27%	2.35%	1.58E-02	1.942
20	Q9Y5T5	USP16	Ubiquitin carboxyl_terminal hydrolase 16	93,570	2	2	3	3	14	3.78%	3.47%	4.88E-02	1.942
21	Q9H7D0	DOCK5	Dedicator of cytokinesis protein 5	215,309	2	2	3	3	7	0.97%	0.89%	3.55E-03	1.941
22	O60476	MAN1A2	Mannosyl_oligosaccharide 1_2_alpha_mannosidase IB	73,004	2	2	2	2	4	3.68%	3.47%	1.41E-02	1.936
23	P28290	SSFA2	Sperm_specific antigen 2	138,386	2	2	4	3	6	2.23%	2.37%	1.10E-03	1.928
24	O60347	TBC1D12	TBC1 domain family member 12	85,626	2	2	2	2	8	3.33%	3.94%	4.63E-03	1.921
25	P53814	SMTN	Smoothelin	99,059	2	2	2	3	6	4.88%	4.68%	1.65E-02	1.916
26	Q96IY1	NSL1	Kinetochore_associated protein NSL1 homolog	32,162	2	2	6	2	11	6.98%	6.16%	5.80E-03	1.91
27	Q8IW19	APLF	Aprataxin and PNK_like factor	56,956	2	3	6	3	8	5.61%	4.85%	4.88E-02	1.909
28	Q9ULV1	FZD4	Frizzled_4	59,881	2	2	17	5	23	12.46%	11.13%	3.15E-03	1.902
29	Q9NZ38	IDI2_AS1	Uncharacterized protein IDI2_AS1	21,312	2	2	2	2	3	7.45%	7.89%	4.59E-02	1.879
30	P14868	DARS	Aspartate__tRNA ligase_ cytoplasmic	57,136	3	3	15	5	19	8.18%	13.77%	7.77E-03	1.847
31	Q9NSE7	ABCC13	Putative ATP_binding cassette sub_family C member 13	30,831	2	2	13	4	17	8.78%	15.61%	2.03E-02	0.548
32	A6NHQ2	FBLL1	rRNA/tRNA 2_O_methyltransferase fibrillarin_ protein 1	34,675	2	2	16	3	28	7.41%	7.19%	2.08E-02	0.538
33	Q6ZNB6	NFXL1	NF_X1_type zinc finger protein NFXL1	101,339	2	2	5	2	7	2.71%	3.35%	4.56E-02	0.538
34	Q96JB2	COG3	Conserved oligomeric Golgi complex subunit 3	94,096	2	2	3	4	11	5.37%	5.37%	4.47E-02	0.533
35	Q9H0G5	NSRP1	Nuclear speckle splicing regulatory protein 1	66,390	2	2	7	3	10	4.19%	5.74%	1.80E-02	0.529
36	Q9ULS6	KCNS2	Potassium voltage_gated channel subfamily S member 2	54,237	2	2	14	3	21	6.65%	8.31%	1.51E-02	0.477
37	O95760	IL33	Interleukin_33	30,759	2	1	2	2	3	6.64%	7.18%	4.34E-02	0.435

### Gene ontology analysis of proteome changes

**Figure 2:**
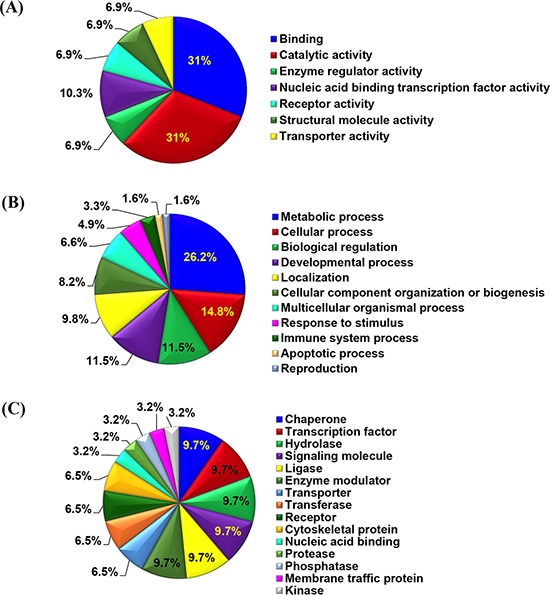
Gene ontology analysis of proteome changes Identified proteins showing > 1.8 fold change were systematized on the basis of **(A)** molecular functions, **(B)** biological processes and **(C)** protein classes, by PANTHER classification system.

To better understand the biological pathways being affected by modulation of protein abundance in response to sanguinarine, the selected 37 proteins were annotated with gene ontology (GO) terms using Protein ANnalysis THrough Evolutionary Relationships (PANTHER) classification system. The distribution of these proteins among molecular functions, biological processes and protein classes are illustrated in Figure 2. It is noteworthy that most of the differentially regulated proteins are involved in binding and catalytic activity, followed by lesser involvement of enzyme regulator, nucleic acid binding transcription factor, receptor, structural molecule and transporter activity (Figure 2A). The GO analysis on the basis of biological processes, which show the molecular events pertinent to the functioning of integrated living system, explored the majorly involvement of proteins related with metabolic and cellular processes (Figure 2B). Sanguinarine also modulate biological regulation, developmental process, localization, cellular component organization, multicellular organismal, response to stimulus, immune system, reproduction and apoptotic processes. The protein class analysis shows the pleotropic mode of action of sanguinarine as it affects broad category of protein classes namely chaperones, transcription factors, hydrolase, signaling molecules, ligase, enzyme modulators, transporter, transferase, receptor, cytoskeletal protein, nucleic acid binding, protease, phosphatase, membrane traffic protein and kinase (Figure 2C). Taken together, the data from gene ontology suggest that sanguinarine affects multiple critical cellular processes that are relevant to cell growth and proliferation.

### Pathway analysis by IPA software

**Figure 3:**
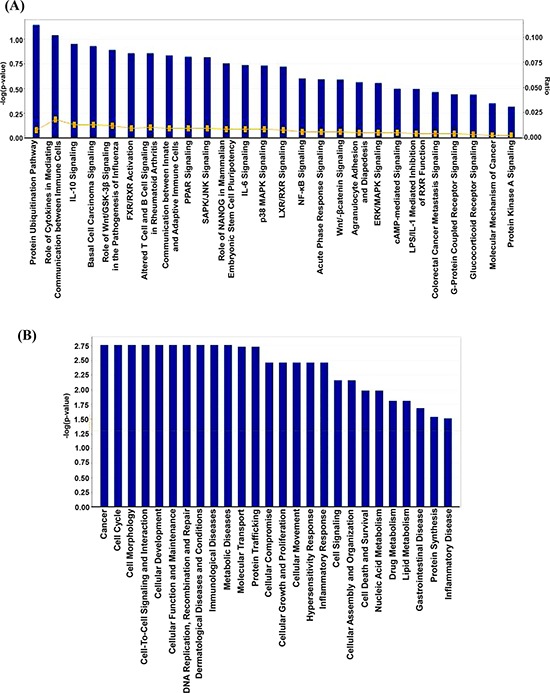
IPA analysis of proteins changing in abundance with sanguinarine treatment **(A)** Association of canonical signaling pathways with modulated proteins are shown. The proteins which demonstrated significant change (95% confidence interval with statistical significance) were subjected to IPA analysis. The top 26 canonical pathways were identified as significantly altered upon sanguinarine treatment. The line bar represents the threshold of significance ( *p* = 0.05). **(B)** IPA was further used to categorize the proteins on the basis of disease and/or functional relation to the altered proteins.

The relationships, putative networks and canonical pathways analysis of differentially expressed proteins were performed by Ingenuity Pathway Analysis (IPA) (Ingenuity Inc.). The selected 37 proteins (Table 1) were uploaded to the IPA module with their corresponding Swiss-Prot IDs and respective fold changes to map proteins into biological networks and to retrieve functions and key pathways. We were able to identify association of 26 canonical pathways with these selected proteins. Among these, the protein ubiquitination pathway which generally involves in post translational modification, appears the top hit. The protein ubiquitination system functions in a variety of cellular processes, including apoptosis, cell cycle and division, DNA transcription and repair, response to stress and extracellular modulators. Further, as shown in Figure 3A, we identified an involvement of several important signaling pathways in the biological response of sanguinarine.

Next, we organized the sanguinarine-modulated protein network into distinct interaction networks, to predict involvement of disease and function-related processes. As shown in Figure 3B, most of these networks account for biological functions related to cancer, cell morphology, cell cycle, cell-to-cell signaling and interaction, cellular development and cellular function and maintenance, and DNA replication, recombination and repair.

**Figure 4:**
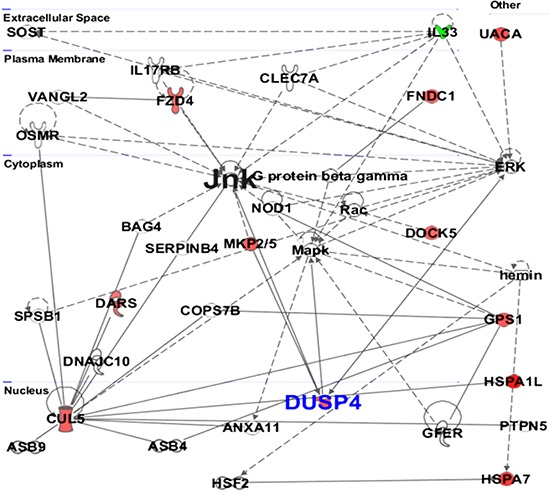
Protein-protein interaction by IPA analysis IPA was further used to determine the protein-protein interactions among modulated proteins. The solid lines denote a robust correlation with partner proteins, and dashed lines indicate statistically significant but less frequent correlations. The upregulated proteins upon sanguinarine treatment are represented in red color whereas the downregulated proteins are shown in green. The un-colored nodes indicate additional proteins of this network that were not spotted by the proteomics analysis. The protein-protein interactions are indicated by arrows.

The protein-protein networks of sanguinarine-modulated proteins were algorithmically generated based on their connectivity. The significance values for network and pathway analyses were computed using Fisher's Exact test. Multiple central nodes, namely L33, ERK, JNK, MAPK, CUL5, GPS1 and DUSP4, were identified from protein-protein networks (Figure 4). However, ERK, JNK and MAPK appeared as additional proteins of this network that were not identified by the proteomics analysis. The protein interaction networks indicated a marked association of DUSP4 in anti-proliferative effects of sanguinarine in pancreatic cancer cells (Figure 4). The protein networks were further systemized through IPA to determine which of the identified proteins are involved in cancer-specific distinct interaction networks. As shown in Supplementary Figure 5, the complex network generated found DUSP4 as an important hub.

### Validation by qRT-PCR analysis

**Figure 5:**
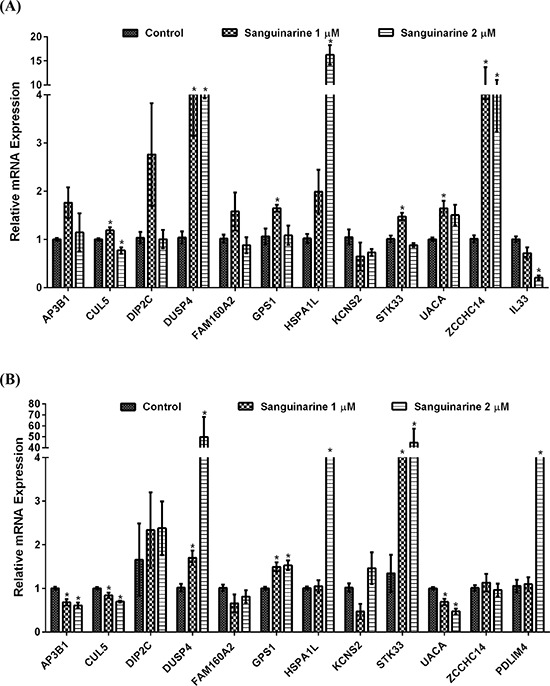
qRT-PCR validation of differentially expressed mRNAs qRT-PCR analysis were performed to validate the protein changes at mRNA levels in sanguinarine-treated **(A)** BxPC-3 and **(B)** MIA PaCa-2 pancreatic cancer cells. cDNA synthesis and PCR assays were carried out as detailed in ‘Materials and Methods’. For each gene, data are shown as S0, cells treated with ethanol; S1, cells treated with sanguinarine at 1 μM; and S2, cells treated with sanguinarine at 2 μM. Data are represented as mean value ± standard errors of minimum three biological replicates, and statistical significance (*p-value < 0.05).

Changes in protein levels can attributed to regulation at multiple levels including transcription, translation, and degradation. To determine if the observed changes were at the transcription level, we performed a qRT-PCR validation of proteomics data. For this purpose, we included only those proteins that showed > 2.0 fold differential expression. Out of 37 proteins, only 18 proteins showed > 2-fold differential expression. This was done to focus on most obvious/major protein modulated by sanguinarine. However, out of these 18, only 13 proteins were detectable and showed consistent results during qRT-PCR validation. This validation was done in two cell lines (BxPC-3 and MIA PaCa-2) at two concentrations of sanguinarine (1 and 2 μM). A minimum of three biological replicates were used for final analysis presented in Figure 5. Overall, we found that most of the modulated proteins identified in the proteomics analysis seems to follow the same trend at the transcription level at sanguinarine 1 μM. However, at the higher concentration of sanguinarine (2 μM), we observed a different trend of mRNA changes that was not consistent between the BxPC-3 and MIA PaCa-2 cells (Figure 5). Contrary to proteomics and mRNA data of BxPC-3 cells, decreasing trend of mRNA level of AP3B1, CUL5, FAM160A2 and UACA were found in sanguinarine treated MIA PaCa-2 cells. IL33 mRNA level in MIA PaCa-2 cells, and PDLIM4 mRNA level in BxPC-3 cells were not detectable. However, DUSP4 seems to follow the same trend as shown by proteomics analysis in both cell lines and shows dose-dependent effects.

### Validation by immunoblot analysis

**Figure 6:**
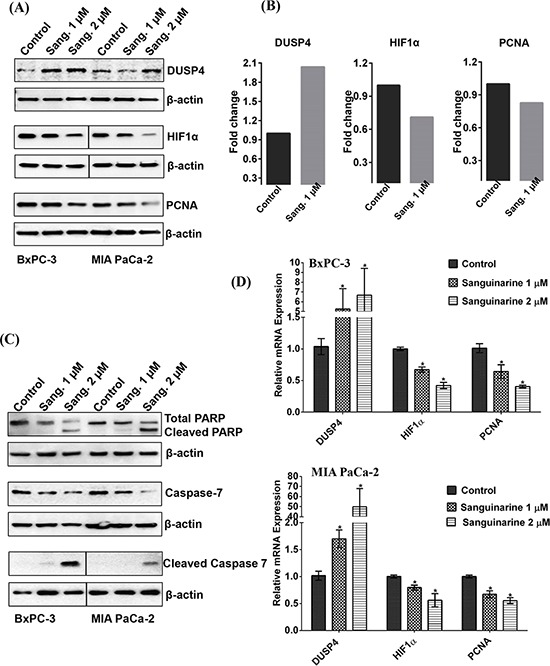
Validation of differentially expressed proteins by immunoblot analysis Immunoblot analyses of vehicle control and sanguinarine-treated samples were performed as detailed in ‘Materials and Methods’. **(A)** Representative immunoblots demonstrating changes in DUSP4, HIF1α and PCNA are shown. **(B)** A re-plot from proteomics data is presented to show changes in DUSP4, HIF1α and PCNA. **(C)** Effect of sanguinarine on cleavage of PARP and Caspase 7 is shown by immunoblot analysis. The blots were re-probed with β-actin for loading control. Results shown are from the same membrane, line denotes removal of a single lane between the two samples. The data are representative of three biological replicates. **(D)** qRT-PCR analysis were performed to validate the protein changes at mRNA levels for HIF1α and PCNA in sanguinarine-treated BxPC-3 and MIA PaCa-2 cells. qRT-PCR data of DUSP4 from Figure 5, are re-plotted with HIF1α and PCNA. For each gene, data are shown as S0, cells treated with ethanol; S1, cells treated with sanguinarine at 1 μM; and S2, cells treated with sanguinarine at 2 μM. Data are represented as mean value ± standard errors of minimum three biological replicates, and statistical significance (*p-value < 0.05).

Although sanguinarine seems to affect several regulatory molecules, the observed effect on DUSP4 was consistent and predominantly visible. Therefore, immunoblot analysis was performed for validation of changes in DUSP4. Further, we also validated two additional relevant proteins, HIF1α and PCNA, due to their well-established association with pancreatic cancer progression. These proteins were found to be downregulated in proteomics data, though at less than 1.8 fold. As shown in Figure 6A, immunoblot analysis revealed an upregulation of DUSP4, and downregulation of HIF1α and PCNA in sanguinarine treated BxPC-3 and MIA PaCa-2 cells in a sanguinarine concentration dependent manner. In the Figure 6B, we have re-plotted the proteomics data obtained for DUSP4, HIF1α and PCNA, for a comparison. We also performed qRT-PCR analysis to assess the mRNA levels for HIF1α and PCNA. We noticed a dose dependent decrease in HIF1α and PCNA mRNA level (Figure 6D). In addition, we determined the effect of sanguinarine on Poly (ADP-ribose) polymerase (PARP) and Caspase 7, which are frequently dysregulated in cancer to allow the cancer cell to evade apoptosis. As shown in Figure 6C, Sanguinarine treatment resulted in an increase in a marked cleavage of PARP and Caspase 7 in both BxPC-3 and MIA PaCa-2 pancreatic cancer cells. To further confirm the effect of sanguinarine on apoptosis induction in BxPC-3 and MIA PaCa-2 cells, we employed flow cytometric analysis using Annexin V/PI binding and CellQuest 3.0 software system. The results showed a dose-dependent increase in cells positive for both Annexin V and PI, indicating an increase in apoptosis (Supplementary Figure 6A and 6B).

## DISCUSSIONS

Despite clinical and technological advances, the rate of fatality due to pancreatic cancer have remained unchanged. This necessitates a need for newer mechanistically driven approaches for treatment of pancreatic cancer. Data amassed from a number of studies has suggested that the plant-alkaloid sanguinarine may have promise in the therapy of cancer, including pancreatic cancer. The Bloodroot plant (*Sanguinaria canadensis*) that contains sanguinarine has a long history of use in folk-medicine, for treatment of variety of illnesses including cancer. An internet search reveals current use of sanguinarine-based modalities for cancer treatment in veterinary patients. In addition, sanguinarine has widely been used in toothpaste, mouthwashes and for other antimicrobial properties [19]. However, these practices are not based on robust scientific studies. Detailed studies are needed to ascertain the efficacy, mechanism, and toxicity of sanguinarine for its potential use as anti-cancer therapeutics in the modern medicine.

In this study, we employed quantitative proteomics to determine the mechanism of anti-proliferative effects of sanguinarine in pancreatic cancer. As described in ‘Results’, our proteomics analysis have revealed that sanguinarine modulates multiple key pathways, which are relevant to cancer growth. We found that a big proportion of the proteins affected by sanguinarine are involved in cellular metabolism. This is an interesting finding because on the way to tumorigenesis and tumor progression, cells undergo metabolic reprogramming to fulfill their energy requirement.

Based on IPA analysis (Figure 3A), protein ubiquitination was identified as the top canonical pathway affected by sanguinarine in our study. In a cell, protein ubiquitination adjusts protein turnover by closely regulating the degradation of protein of interest. The proteins identified by IPA analysis that are directly involved in ubiquitin/proteasome pathways include DUSP4, USP16 and HSPA7. Interestingly, Cagnol and Rivard have shown that DUSP4 becomes ubiquitinated and degraded through proteasome, and proteasome inhibition increases basal DUSP4 expression in non-stimulated BRAFV600E:ER cells [20]. The disease and functions canonical pathways of the altered proteins shows that sanguinarine targets a variety of cancer related processes (Figure 3B). Further, multiple central nodes (CUL5, IL33, GPS1 and DUSP4) were identified in IPA analysis to be affected by sanguinarine (Figure 4). These newly identified ‘central nodes’ are very interesting and relevant to cancer development and progression.

Cullin-5 (CUL5) is known to inhibit cellular proliferation. Fay and colleagues have found a decrease level of CUL5 in human breast tumor tissue supporting its potential role in breast tumorigenesis [21]. In this study, we found increased level of CUL5 at the proteomics level and mRNA level in BxPC-3 cells (Table 1, Figure 5A). Interleukin-33 (IL-33) is a pro-inflammatory molecule that has been suggested to act as a crucial mediator in inflammation-associated pancreatic carcinogenesis [22]. Our proteomics data as well as qRT-PCR data shows the marked decrease in the expression of IL-33 (Table 1, Figure 5A). In this study, we found a significant increase in G protein pathway suppressor 1 (GPS1) by sanguinarine. GPS1 suppresses G-protein- and mitogen-activated protein kinase-mediated signal transduction [23]. However, there is no reported role for GPS1 in pancreatic cancer at present. Our study also identified the Dual specificity phosphatase 4 (DUSP4) as a novel protein modulated by sanguinarine. DUSP4 dephosphorylate both Thr and Tyr residues on MAP kinases (ERK1 and ERK2) to regulate the mitogenic signal transduction [24]. DUSP4 shows higher substrate specificity *in vitro* for ERK followed by JNK and P-38, respectively [24, 25]. Interestingly, oral supplementation of sanguinarine has been shown to inhibit tumor burden in A375 human melanoma in athymic nude mice via inhibiting the activation of mitogen-activated protein kinases (p-p44/42 MAPK) and protein kinase B (pAKT) [26]. Niu and colleagues have shown that the anti-inflammatory effects of sanguinarine are mediated by inhibition of p38 MAPK and ERK1/2 phosphorylation [27]. Furthermore, the protein network data of sanguinarine-modulated proteins show that DUSP4 negatively regulates ERK, JNK, MAPK. Our proteomics data followed by qRT-PCR and immunoblot analysis demonstrated that sanguinarine significantly increases DUSP4 in pancreatic cancer BxPC-3 and MIA PaCa-2 cells. The role of DUSP4 in pancreatic cancer has not yet been explored; however, an association of DUSP4 loss with cancer progression has been shown in certain cancers [28, 29]. Balko and colleagues found that the loss of DUSP4 activates MAPK pathways which in turn stimulates cancer stem cell-like phenotypes in basal-like breast cancer [29]. Recently, Waha *et al*. have found frequent epigenetic downregulation of DUSP4 in glioma [30]. Furthermore, exogenous overexpression of DUSP4 has been shown to inhibit glioblastoma cell growth, indicating DUSP4 as a possible tumor suppressor [30]. Our study suggests that DUSP4 is an important central hub, which interacts with several important molecules modulated by sanguinarine in pancreatic cancer.

In our study, we found that sanguinarine treatment also resulted in a marked cleavage of PARP and Caspase 7, which are indicative of apoptosis induction (Figure 6C). We further confirmed sanguinarine induces apoptosis using Annexin V/Propidium Iodide (PI) binding assay (Supplementary Figure 6A and 6B). This data confirms our previous study where we demonstrated apoptosis-induction by sanguinarine in BxPC-3 and AsPC1 cells [9]. Further, the immunoblot analysis also showed that sanguinarine causes a decrease in hypoxia-inducible factor 1 alpha (HIF1α) and proliferating cell nuclear antigen (PCNA) in both BxPC-3 and MIA PaCa-2 pancreatic cancer cells (Figure 6A). We also found a significant decrease in mRNA level of HIF1α and PCNA as a result of sanguinarine treatment in these cells (Figure 6D). While PCNA is a marker of cellular proliferation, HIF1α has been shown to be associated with negative prognosis of several cancers including pancreatic cancer. In fact, hypoxia is known to alter cellular metabolism (by transcriptional activation of genes) and promote neoangiogenesis that leads to sustained tumor growth and survival [31]. Thus, it appears that sanguinarine decreases cellular hypoxia and cell proliferation in addition to apoptosis induction that leads to inhibition of pancreatic cancer cells.

We have previously demonstrated that BCL-2 family proteins and p53 play the key roles in the biological effects of sanguinarine in pancreatic cancer cells [9]. Specifically, we found that sanguinarine treatment resulted in a slight decrease in p53 protein level and a significant increase in its phosphorylated form [9]. In our current investigations, p53 appeared in the proteomics data with negligible change (Supplementary Table 1), however, phosphorylated p53 and BCL-2 didn't appear as visible proteins in criteria selected for quantitative proteomics analysis. Indeed, studies have shown associations between p53, BCL-2 and DUSP4. While the tumor suppressor p53 is a well-known regulator of BCL-2 (based on a number of studies), Shen and colleagues have shown that DUSP4 is a novel transcription target of p53 [32]. Interestingly, Shen and colleagues have also shown that DUSP4 can induce apoptosis even in the absence of p53, thus highlighting the p53-independent role of DUSP4 in apoptosis signaling [32].

Taken together, our study has suggested a pleotropic nature of sanguinarine as it negatively regulates multiple pathways relevant to pancreatic cancer cell survival. Indeed, studies suggest that pancreatic cancer is a genetically complex and heterogeneous disease. A recent study has suggested that the mature pancreatic cancer cell carries on average 63 genetic alterations which is a core panel of 12 cellular signaling pathways and processes that affect 67–100% of the tumors [33]. Therefore, a successful drug for pancreatic cancer management will have to affect multiple targets to attack heterogeneous population of pancreatic cancer cells. Our study has shown that sanguinarine possesses these characteristics. In addition to the pathways identified by our study, sanguinarine has been shown to modulate several other important pathways. For example, sanguinarine has been shown to facilitate the formation and increase the stabilization of DNA G-quadruplex, which could potentially damage cancer cells [15]. Importantly, sanguinarine appears to have a reasonable safety profile as it was found to be safe at a dose of 5 mg/kg body weight in pigs [34]. In addition, we have previously shown that sanguinarine doesn't affect normal human epidermal keratinocytes at up to 2.0 μM concentration [10]. Similarly, Sun and colleagues have shown that 1 μM sanguinarine does not have any effect on the growth of immortalized normal prostate epithelial cells [35]. However, further detailed studies, especially in relevant *in vivo* models, are needed to further ascertain the usefulness and efficacy of this promising herbal agent against pancreatic cancer.

## MATERIALS AND METHODS

### Cell culture and treatments

BxPC-3 and MIA PaCa-2 human pancreatic cancer cells (ATCC) were cultured in Dulbecco's Modified Eagle's Medium (Corning Cellgro) supplemented with 10% fetal bovine serum (Sigma). Cells were maintained in a humidified incubator with 5% CO_2_ at 37°C. BxPC-3 and MIA PaCa-2 cells were authenticated at University of Wisconsin, Translational Research Initiatives in Pathology laboratory (TRIP Lab) by STR Analysis. This was done using the Promega PowerPlex 16 HS System kit (DC2101), which analyzes 15 STR loci and one sex determinant. Before experimentation, these cells were tested for mycoplasma using the Mycoplasma Detection Kit (Lonza) according to manufacturer's protocol, with fluorescence readings done on a Synergy H1 Multimode microplate reader (BioTek). Sanguinarine (≥98% purity) was purchased from Sigma. Stock concentration of sanguinarine (5 mM) was made in ethanol. The cells were treated with sanguinarine as described later. Ethanol alone treatment served as the control.

### MTT, trypan blue exclusion- and clonogenic cell survival assays

The effect of sanguinarine on cell proliferation against BxPC-3 and MIA PaCa-2 cells were analyzed using an 3-(4,5-dimethylthiazol-2-yl)-2,5-diphenyltetrazolium bromide (MTT) assay. Briefly, 10,000 cells were seeded in 96-well plate, next day treated with sanguinarine (0.1, 0.2, 0.5, 1.0, 2.0, 4.0 and 8.0 μM; for 24 and 48 h), followed by incubation with MTT reagent for 2 h. MTT formazan crystals were dissolved in DMSO and optical density was measured at 570 nm on a Synergy H1 Multimode microplate reader (BioTek). The effect of sanguinarine treatments on the growth and viability of BxPC-3 and MIA PaCa-2 cells was assessed by Trypan blue exclusion assay. Briefly, following sanguinarine treatment, the cells were collected and stained with Trypan blue dye (which specifically stains non-viable cells), and counted using a Bio-Rad cell counter (Bio-Rad Laboratories Inc.). For clonogenic cell survival, following treatment of cells with sanguinarine, cells were collected by trypsinization, counted and re-plated equal number of cells at low density (3,000 cells) in triplicate in six-well tissue culture plates (TPP). The cells were allowed to grow for 14 days followed by staining with crystal violet. Images of cell colonies were procured using a digital scanner.

### Annexin V/Propidium Iodide (PI) binding assay for apoptosis

Apoptosis was measured using a VybrantTM apoptosis assay kit (Molecular Probes) as per as manufacturer's protocol. Briefly, BxPC-3 and MIA PaCa-2 cells (10^5^) were seeded in 6-well plates and next day, treated with 1.0 and 2.0 μM of sanguinarine for 24 h. After treatment, cells were trypsinized and resuspended in Annexin V binding buffer followed by incubation with Annexin V-FITC for 15 min at 4°C in the dark. Cells were further stained with 5 μl of PI for another 5 min at 4°C in the dark. Apoptotic cells were analyzed immediately on a flow cytometer (BD Biosciences) with the CellQuest 3.0 software system.

### Sample preparation and LC/MS/MS analysis

For quantitative proteomics analysis, BxPC-3 cells were treated with vehicle or sanguinarine (1 μM) for 24 h. The treatments were done in duplicate and the experiment was repeated three times to yield a total of 6 replicates (3 biological replicates X 2 technical replicates). Treated cells were collected by trypsin digestion followed by centrifugation and washing with PBS to obtain cell pellets, which were stored at −80°C for nano-LC/MS/MS.

Protein preparation and nano-LC/MS/MS were performed at School of Pharmacy Analytical Instrumentation Center Mass Spectrometry facility. A schematic representation of the process is provided in Figure 1A. Briefly, proteins were extracted from the frozen cell pellets after addition of 0.3 ml ice cold PBS by passing them through a 23 gauge needle 10–15 times. Cell lysates were then cleared by centrifugation at 10,000 g for 10 min at 4°C. Protein concentration of the extracts was then determined by Micro BCA (Thermo Fisher Scientific/Pierce). Sample protein (20 μg) was digested with 1 μg sequencing grade trypsin (Promega Corp.). Following an overnight digestion, samples were prepared for LC/MS/MS by C18 Zip-Tip purification according to the manufacturer's protocol (Millipore Inc.). Samples were then suspended in water with 0.1% formic acid (v/v) and subjected to nano-LC/MS/MS.

For LC/MS/MS, the samples were analyzed by injecting 1 μg of the digest onto a reverse phase BEH C18 column (100 μm x 100 mm), with 1.7 μm, 300 Å pore particles size (Waters Corp. Milford) using a Waters nanoACQUITY chromatography system. Peptides were eluted from the column using a 180 min increasing organic gradient. Solvent A was water/0.1% formic acid (v/v), while solvent B was acetonitrile/0.1% formic acid (v/v). The gradient started at 3% B and increased with a linear gradient to 35% B at 130 min. At 140 min the gradient increased to 95% B and held for 10 min. At 160 min the gradient returned to 3% to re-equilibrate the column for the next injection. Peptides eluting from the column were analyzed by data-dependent MS/MS on a Q-Exactive Orbitrap mass spectrometer (Thermo Fisher Scientific Inc.). A top 15 method was used to acquire data. The instrument settings were as follows: the resolution was set to 70,000, the AGC target was set to 10^6^ counts, the scan range was from 300–2000 m/z, the MS scan was recorded in profile while the MS/MS was recorded in centroid mode, dynamic exclusion was set to 25 seconds.

### Data processing and protein identification by human database search

Following LC/MS/MS acquisition, the data were searched against the Swiss-Prot human proteome database with decoy using Sequest HT search engine under the Proteome Discoverer 1.4 software (Thermo Fisher Scientific Inc.). Proteins were identified at a false discovery cut off < 1%. Following protein identification the LC/MS/MS data were aligned using Chromalign software. Quantitation of peptides eluted between 30 and 130 min were performed on the processed data using SIEVE 2.1 (Thermo Fisher Scientific Inc.). The protein list (as detailed in Supplementary Table 1) were generated with set *p*-value 0.05 for the identified proteins. Data were filtered to remove all proteins identified by only a single peptide (no one-hit wonders). Additional filtering of the data was done to remove peptides with a coefficient of variance (CV) > 30% in the 6 replicates.

### Pathway analysis

To understand pathways modulated by sanguinarine, a list of differentially expressed (> 1.8 fold) was compiled. These proteins were categorized according to their Gene ontology (GO) descriptions using information from the GO database and PANTHER (Protein ANnalysis THrough Evolutionary Relationships; http://www.pantherdb.org/) classification systems. The proteins were projected on the basis of their molecular functions, biological processes and protein classes.

The canonical pathways, disease/function pathways and protein-protein interactions were analyzed using Ingenuity Pathway Analysis Software (IPA trial version, Ingenuity Systems, http://www.ingenuity.com) (Qiagen) [36]. The predicted protein-protein interaction networks and canonical pathways were generated using inputs of gene identifiers, log2 fold-changes and *p*-values between control and treated group comparisons.

### Real-time PCR analysis

Quantitative reverse-transcriptase polymerase chain reaction in real time (qRT-PCR) was used for validation modulated proteins at the transcription level. QIAshreder and RNeasy Mini Kit (Qiagen) were used to isolate total RNA from the cells following treatments, as per the manufacturer's protocol. The first strand cDNA was transcribed with random primers, dNTPs and M-MLV reverse transcriptase (Promega). qRT-PCR was performed using the StepOnePlus Real-Time PCR system (Applied Biosystems/Life Technologies Corp.) and SYBR Premix Ex Taq II (TaKaRa) with first strand cDNA, forward and reverse primers. Primers for STK33, HSPA1L, FAM160A2, AP3B1, UACA, PDLIM4, ZCCHC14, GPS1, DIP2C, DUSP4, CUL5, KCNS2, IL33, PCNA and GAPDH were selected from the PrimerBank database [37]. HIF1α primers used are described elsewhere [38]. All the primer sequences used in this study are listed in Supplementary Table 2. The PCR program was set as: initial denaturation step (95°C for 30 s) followed by DNA amplification (95°C for 3 s followed by 61°C for 30 s) for 40 cycle. Melt curve analysis was performed to ensure the specificity of target amplicon. A housekeeping gene, GAPDH was used for normalization and ∆∆CT algorithm was used for relative quantification of target amplicons.

### Immunoblot analysis

A subset of proteins identified in LC/MS/MS analysis (DUSP4, HIF1α and PCNA) and proteins related with apoptosis (PARP and Caspase 7) were selected for further validation by immunoblot analysis. Briefly, following treatments, cells were collected and lysed in 1X RIPA buffer (EMD Millipore Corp.) containing protease inhibitor cocktail (Thermo Fisher Scientific Inc.) and 1 mM PMSF (Amresco LLC). The protein concentration was determined by the BCA Protein Assay (Thermo Fisher Scientific Inc.). For immunoblot analysis, 40 μg proteins were subjected to SDS-PAGE, transferred on to a nitrocellulose membrane, and incubated for 1 h in blocking buffer (5% milk). 1X TBST buffer was used for blot washing as well as to make 5% milk. The membranes were probed with DUSP4 (Abnova Corporation), HIF1α, PCNA and Caspase 7 (Santa Cruz Biotechnology Inc.), PARP and β–actin (Cell Signaling) primary antibodies followed by appropriate secondary horseradish peroxidase (HRP)-conjugated antibodies. The proteins were detected by enhanced chemiluminescence (Thermo Fisher Scientific Inc.) and HRP enzyme activity. The images of immunoblots were captured by Kodak Image Station 4000MM (Carestream Health Inc.). Re-probing with β–actin served as loading control of protein samples.

### Statistical analysis

The LC-MS/MS data from 6 control and 6 sanguinarine treated samples were analyzed using multiple software like Chromalign, SIEVE 2.1 and IPA as detailed above. The qRT-PCR data were analyzed using StepOne Software v2.2 RQ Study (Applied Biosystems/Life Technologies Corp.) and exported as RQ_max_ and RQ_min_ (2^−∆∆Ct +/− ∆∆Ct SD^) which represent relative quantity for gene of interest. RQ_max_ and RQ_min_ are the results of incorporating standard deviation of the ∆∆CT into the fold change calculations. Further, statistical analyses on biological replicates of qRT-PCR data were performed with GraphPad Prism 5 software (GraphPad Software Inc.) using one-way analysis of variance (ANOVA) followed by Dunnett's multiple comparison test.

## SUPPLEMENTARY FIGURES AND TABLE




