# Supplementary material for: Molecular signatures of sanguinarine in human pancreatic cancer cells: A large scale label-free comparative proteomics approach
Source: Oncotarget. 2015 Apr 13;6(12):10335–49. doi: 10.18632/oncotarget.3231 (PMC4496359; doi:10.18632/oncotarget.3231)
Supplement: Supplementary file 1 [file oncotarget-06-10335-s001.pdf]

SUPPLEMENTARY FIGURES AND TABLE

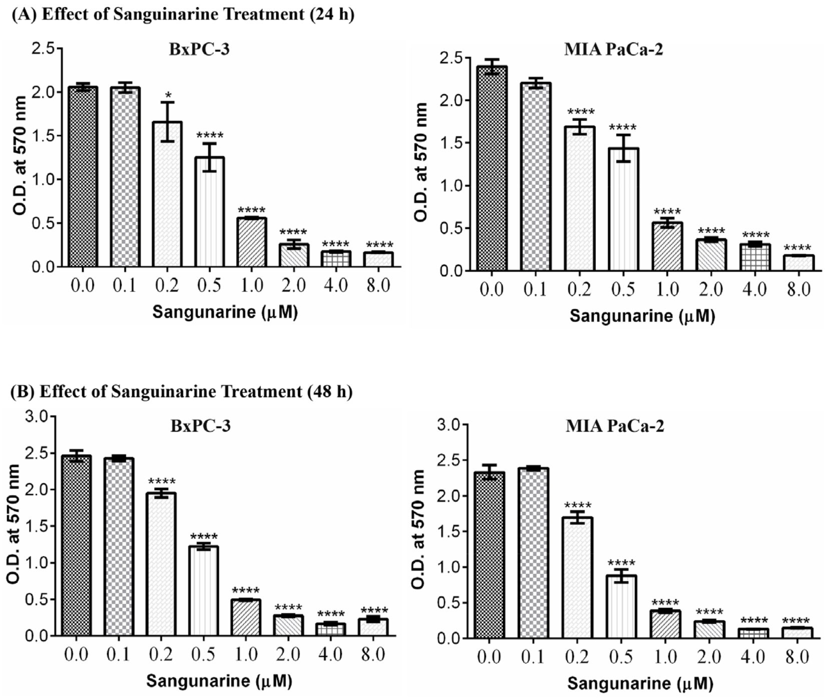

Supplementary Figure 1: Effect of sanguinarine on cell proliferation (MTT assay) in BxPC-3 and MIA PaCa-2 human pancreatic cancer cells. BxPC-3 and MIA PaCa-2 cells were treated with different concentration of sanguinarine (0.1, 0.2, 0.5, 1.0, 2.0, 4.0 and 8.0 μM) for **(A)** 24 h, and **(B)** 48 h. Cell proliferation was assessed by MTT assay. Data are expressed as mean ± standard error of three replicates, and statistical significance are denoted as $*P < 0.05$ and $****P < 0.0001$.

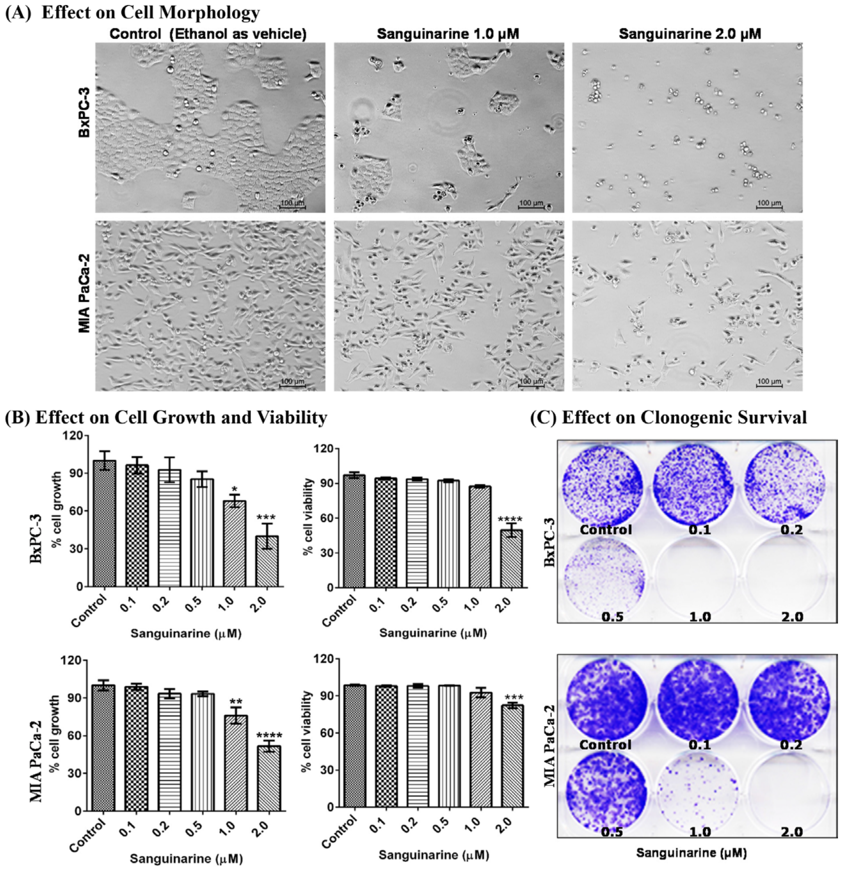

Supplementary Figure 2: Anti-proliferative responses of sanguinarine in BxPC-3 and MIA PaCa-2 human pancreatic cancer cells. **(A)** Effect of sanguinarine (1.0 or 2.0 μM; 24 h) treatment on morphology of BxPC-3 and MIA PaCa-2 cells was analyzed by Nikon Ti Microscope (Nikon Corp., USA) and imaged at 20x. Representative image from each treatment groups are presented. **(B)** Effect of sanguinarine (0 – 2.0 μM; 24 h) treatment on growth and viability of BxPC-3 and MIA PaCa-2 cells was determined by Trypan Blue Exclusion assay using BioRad cell counter. **(C)** Effect of sanguinarine (0 - 2.0 μM; 24 h) treatment on clonogenic survival of BxPC-3 and MIA PaCa-2 cells was assessed. Equal number of treated cells (3,000) were allowed to grow for 14 days followed by staining with crystal violet. The images were captured using a color photo scanner. Cell growth and viability data are presented as mean ± standard error of three replicates and statistical significance are denoted as $^{*}P < 0.05$, $^{**}P < 0.01$, $^{***}P < 0.001$ and $^{****}P < 0.0001$.

(A) Base Peak Alignment

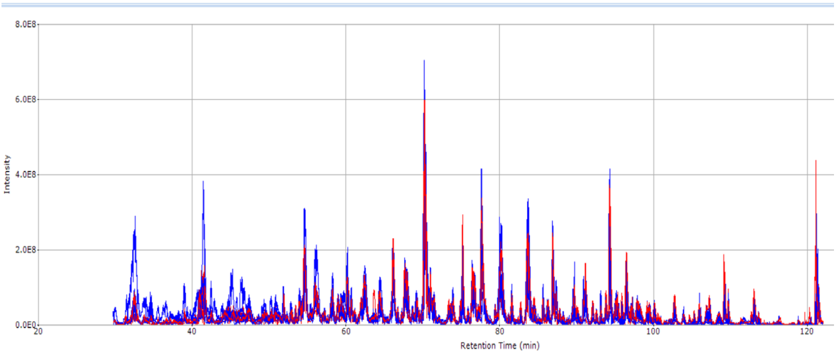

(B) Alignment Scores

File Name	Score
BxPC-3 Control 1	0.886
BxPC-3 Control 2	1.000
BxPC-3 Control 3	0.927
BxPC-3 Control 4	0.908
BxPC-3 Control 5	0.898
BxPC-3 Control 6	0.849
BxPC-3 Treated 1	0.907
BxPC-3 Treated 2	0.859
BxPC-3 Treated 3	0.927
BxPC-3 Treated 4	0.916
BxPC-3 Treated 5	0.934
BxPC-3 Treated 6	0.903

Supplementary Figure 3: Base peak chromatogram alignment using SIEVE 2.1 Chromalign. Data files were loaded into SIEVE 2.1 and the base peak chromatograms were aligned with Chromalign. **(A)** The twelve aligned base peak chromatograms of control and sanguinarine treated samples are shown. **(B)** The alignment scores of control and treated samples presented are generated by Chromalign. SIEVE requires an alignment score of 0.75 or above for quantitation. Scores above 0.85 is considered very good alignment.

(A) Fold Change Breakdown of Identified Proteins

Fold Change	Identified Proteins at 0.05 Confidence Interval
>2.0x	16
<2.0x - >1.5x	45
<1.5x - >1.0x	723
<1.0 - >6.7x	2234
<0.67 - >0.50	85
<0.50	2
Total Identified Proteins:	3107

(B) Peptide Overlap

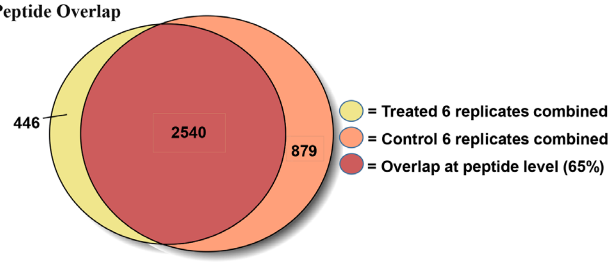

(C) Protein Overlap

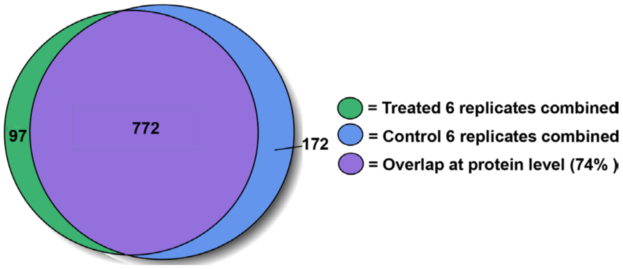

Supplementary Figure 4: Overlap between replicate injections. LC/MS/MS results were searched against the Swiss-Prot human proteome database using Sequest HT run under the Proteome Discoverer software. **(A)** The identified proteins were categorized into various classes of regulation based on the ratios of treated vs. control. **(B)** and **(C)** Venn diagrams illustrating the overlap at peptide and protein level respectively, between control and sanguinarine treated cells are shown.

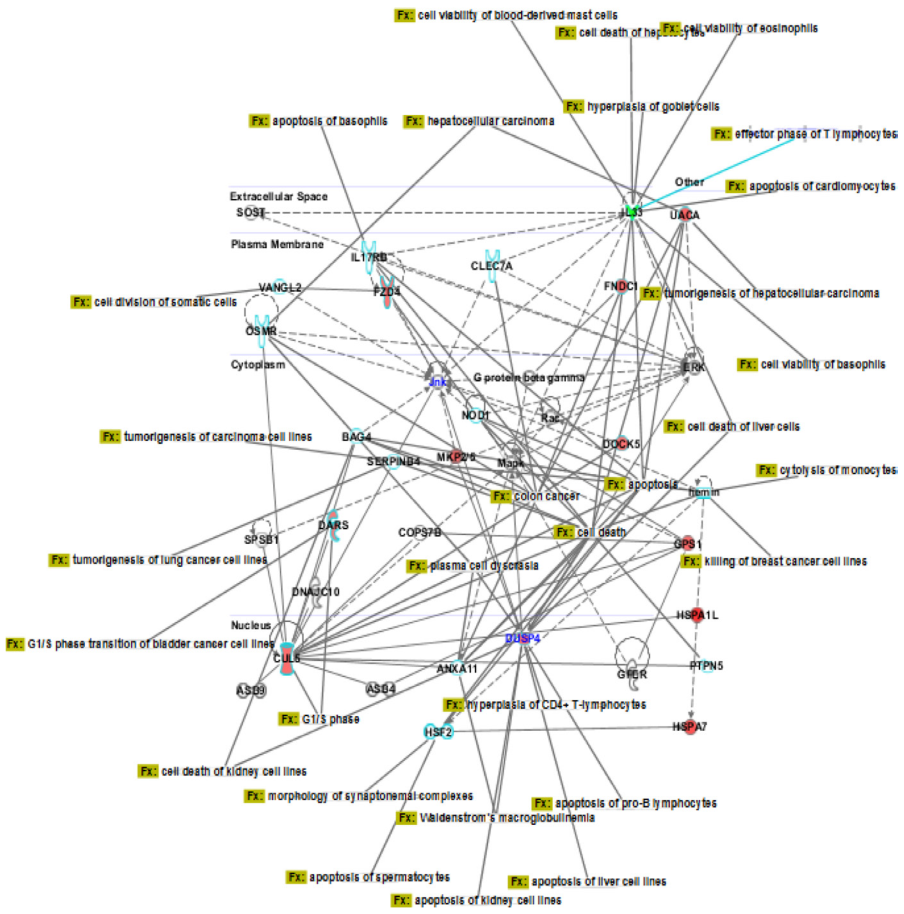

Supplementary Figure 5: Complex networks associated with proteins identified. The proteins were analyzed by IPA to reveal their functional association with various cancers.

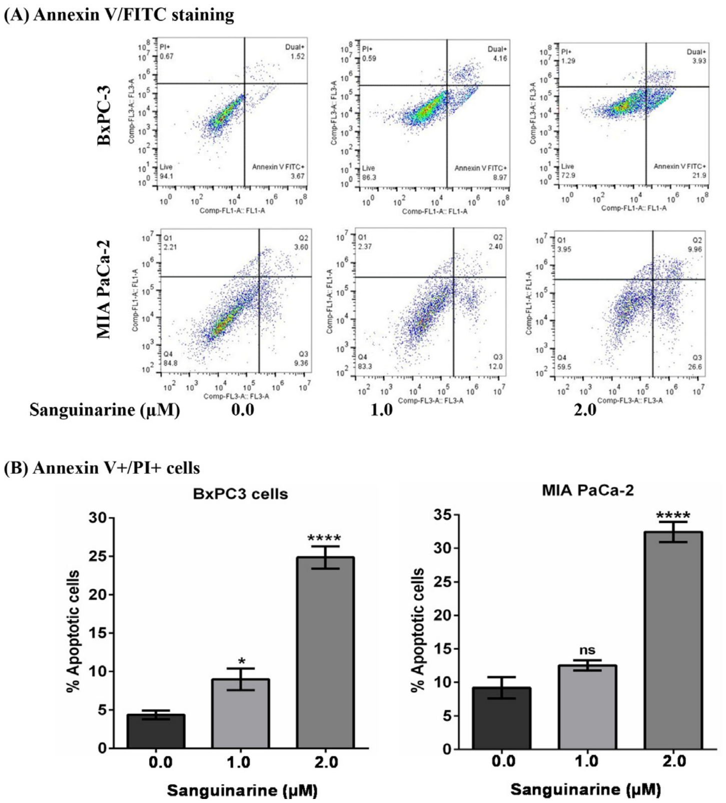

Supplementary Figure 6: Effect of sanguinarine on apoptosis in BxPC-3 and MIA PaCa-2 human pancreatic cancer cells. Effect of sanguinarine (1.0 or 2.0 µM; 24 h) treatment on apoptosis was determined by Annexin V/Propidium Iodide (PI) binding assay. Following treatment, the cells were washed with PBS and stained with FITC conjugated Annexin V antibody and PI as detailed in 'Materials and Methods'. **(A)** Representative 2-dimensional dot plots of Annexin V-FITC and PI fluorescence are displayed for each treatment group. **(B)** Total Annexin V+/PI+ cells representing % apoptotic cells at each treatment level are plotted. Data are presented as mean ± standard error of four replicates and statistical significance are denoted as $*P < 0.05$ and $****P < 0.0001$.

Supplementary Table 1: Complete list of proteins identified by nanoLC-MS/MS analysis

Supplementary Table 2: Primer sequences used for qRT-PCR validation

Gene	Amplicon size (bp)	Primer orientation	Primer Sequence (5' - > 3')	Length	Tm	Location
STK33	188	Forward	GGAAACAGAAACGAAGTGGGC	21	61.7	408–428
		Reverse	CTCCATCCTCACAAAGCTCCA	21	61.5	595–575
HSPA1L	227	Forward	CTACTGCCAAGGGAATCGCC	20	62.7	5.–24
		Reverse	GCCGATCAGACGTTTAGCATCA	22	62.7	231–210
FAM160A2	229	Forward	GGTCCAGTTCGTGAGGCTC	19	62	445–463
		Reverse	TGCCCTCTCGATGCACAAAA	20	62.1	673–654
AP3B1	132	Forward	GAAGCGGATTGTTGGGATGAT	21	60.4	189–209
		Reverse	TCAGCATATCGAACCAGGTAAAC	23	60.1	320–298
UACA	248	Forward	CAATCCAGGCAAACTAGATGTGG	23	61.2	138–160
		Reverse	CGGCATCGTGAAGTGCAGT	19	62.9	385–367
PDLIM4	435	Forward	GCTGCCACGATCACCTCAC	19	63	212–230
		Reverse	CCTGCAAGTAGCGGAAGGA	19	61.4	646–628
ZCCHC14	240	Forward	CGGACGCATTTTATGTGGAGC	21	62	488–508
		Reverse	TCTGCGAGGACGGGATACC	19	62.8	727–709
GPS1	181	Forward	CCCAGGTGCAGAATATCTTGC	21	60.8	683–703
		Reverse	ACTAAGGCTACTAGCTCAGGGT	22	61.5	863–842
DIP2C	121	Forward	TGGACAAGAAGTATGAAGGTCGC	23	62.2	1066–1088
		Reverse	AGGCAGCCGGATCATTGTTG	20	62.9	1186–1167
DUSP4	146	Forward	GGCGGCTATGAGAGGTTTTCC	21	62.8	433–453
		Reverse	CAGCTACATGCAATCTGGCT	20	60.2	1546–1527
CUL5	170	Forward	TCCAAGATAGTGCAATGAAGCTG	23	60.6	464–486
		Reverse	CTCTGTTGAATCCAAGTATGCCT	23	60	633–611
KCNS2	147	Forward	CGCCTCCAAGTTCGATGGG	19	62.7	471–489
		Reverse	AGGCACATGGTGATGATGGAC	21	62.2	617–597
IL33	94	Forward	GTGACGGTGTTGATGGTAAGAT	22	60	143–164
		Reverse	AGCTCCACAGAGTGTTCCTTG	21	61.7	236–216
HIF1α	113	Forward	AGTGTACCCTAACTAGCCGAGGAA	24	60.34	*Ref. 38*
		Reverse	CTGAGGTTGGTTACTGTTGGTATCA	25	60.14	
PCNA	209	Forward	ACACTAAGGGCCGAAGATAACG	22	61.8	265–286
		Reverse	ACAGCATCTCCAATATGGCTGA	22	61.2	473–452
GAPDH	116	Forward	TGTGGGCATCAATGGATTTGG	21	60.9	231–251
		Reverse	ACACCATGTATTCCGGGTCAAT	22	61.4	346–325
